# EEG microstate changes during hyperbaric oxygen therapy in patients with chronic disorders of consciousness

**DOI:** 10.3389/fnins.2023.1145065

**Published:** 2023-04-12

**Authors:** Jiameng Wang, Long Xu, Qianqian Ge, Lianbi Xue, Yaling Liu, Cong Wang, Yue Wu, Yun Liu, Lichao Chen, Yutong Zhuang, Xiaoli Geng, Xueling Chen, Bo Wang, Qiuhong Yu, Jianghong He, Xudong Zhao

**Affiliations:** ^1^AHU-IAI AI Joint Laboratory, Anhui University, Hefei, China; ^2^Hefei Comprehensive National Science Center, Institute of Artificial Intelligence, Hefei, China; ^3^Department of Neurosurgery, Beijing Tiantan Hospital, Capital Medical University, Beijing, China; ^4^China National Clinical Research Center for Neurological Diseases, Beijing, China; ^5^Department of Hyperbaric Oxygenation, Beijing Tiantan Hospital, Capital Medical University, Beijing, China; ^6^Department of Hyperbaric Oxygen, Liaocheng People’s Hospital, Liaocheng, China; ^7^State Key Laboratory of Brain and Cognitive Science, Institute of Biophysics, Chinese Academy of Sciences, Beijing, China; ^8^University of Chinese Academy of Sciences, Beijing, China

**Keywords:** disorders of consciousness, hyperbaric oxygen, electroencephalogram, microstate, clinical assessment

## Abstract

Hyperbaric oxygen (HBO) therapy is an effective treatment for patients with disorders of consciousness (DOC). In this study, real-time electroencephalogram (EEG) recordings were obtained from patients with DOC during HBO therapy. EEG microstate indicators including mean microstate duration (MMD), ratio of total time covered (RTT), global explained variance (GEV), transition probability, mean occurrence, and mean global field power (GFP) were compared before and during HBO therapy. The results showed that the duration of microstate C in all patients with DOC increased after 20 min of HBO therapy (*p* < 0.05). Further statistical analysis found that the duration of microstate C was longer in the higher CRS-R group (≥8, 17 cases) than in the lower group (<8, 24 cases) during HBO treatment. In the higher CRS-R group, the transition probabilities from microstate A to microstate C and from microstate C to microstate A also increased significantly compared with the probability before treatment (*p* < 0.05). Microstate C is generally considered to be related to a salience network; an increase in the transition probability between microstate A and microstate C indicates increased information exchange between the auditory network and the salience network. The results of this study show that HBO therapy has a specific activating effect on attention and cognitive control in patients and causes increased activity in the primary sensory cortex (temporal lobe and occipital lobe). This study demonstrates that real-time EEG detection and analysis during HBO is a clinically feasible method for assessing brain function in patients with DOC. During HBO therapy, some EEG microstate indicators show significant changes related to the state of consciousness in patients with chronic DOC. This will be complementary to important electrophysiological indicators for assessing consciousness and may also provide an objective foundation for the precise treatment of patients with DOC.

## 1. Introduction

Disorders of consciousness (DOC) are states of loss of consciousness caused by various severe brain injuries, such as coma, vegetative state (*VS*), and minimally conscious state (MCS). Prolonged DOC (pDOC) is defined as disorders of consciousness with loss of consciousness for more than 28 days (Giacino et al., 2018; Kondziella et al., 2020). One of the greatest difficulties in DOC graded diagnosis is identifying and differentiating patients who retain some degree of consciousness from those who do not. In the absence of a better way to assess a person’s level of consciousness, physicians can only infer a patient’s state based on the patient’s ability to perform apparently voluntary actions that suggest consciousness. With the development of diagnostic techniques for the level of consciousness, the continuous revision of the Coma Recovery Scale-Revised (CRS-R), and the use of functional brain imaging techniques, which solve the problem of consciousness detection in patients after severe brain injury, our understanding of the classification of DOC is also advancing (Porcaro et al., 2022).

There are many clinical causes of DOC, including traumatic brain injury, cerebrovascular disease, hypoxic ischemic encephalopathy, brainstem injury, and many other causes (Pauli et al., 2020; Zhang et al., 2023). Clinical intervention treatment of DOC is very important for the recovery of consciousness. Insufficient appropriate intervention treatment may lead to a persistent vegetative state, which not only brings a heavy burden to society and families but also causes family members and medical staff to face ethical dilemmas (Schnakers et al., 2009). Accurate identification of UWS/*VS* and MCS is of great significance to the treatment of patients and the selection of medical resources. However, the current clinical misdiagnosis rate of MCS as UWS/*VS* is 41% (Schnakers et al., 2009; Ye et al., 2020). When the patient’s level of consciousness shifts from UWS/*VS* to MCS, there is an inflection point in electroencephalography (EEG) features (Lei et al., 2022). An increasing number of studies have confirmed the role of hyperbaric oxygen (HBO) therapy in the process of awakening in patients with DOC (Ye et al., 2020; Porcaro et al., 2022), and quantitative EEG (qEEG) can be applied to evaluate the effect of the HBO therapy course or intervention time on the curative effect of patients with cerebral resuscitation (Ye et al., 2020). Then, as a therapeutic stimulus, HBO may help to accurately identify the patient’s level of consciousness if the EEG changes during HBO therapy can be monitored, which in turn guides treatment without wasting medical resources or delaying patients’ treatment. However, real-time EEG monitoring during HBO therapy in patients with impaired consciousness has not been reported.

EEG is a technology that analyzes the physiological electrical signal activity of each brain region by recording the electric potential of the scalp surface electrode and the electric field strength (von Wegner and Laufs, 2018). EEG, with a history of nearly a century, has the advantages of low price and high temporal resolution and can noninvasively evaluate the neural activity of brain regions (Plum and Posner, 1972). At present, there are some relatively mature methods to extract effective information from multichannel EEG data, and microstate analysis is one of them (Michel and Koenig, 2018). Lehmann et al. (1987) proved for the first time that the alpha frequency band (8–12 Hz) of the multichannel resting-state EEG signal could be decomposed into a series of quasi-steady states. These discrete states are defined as “microstates,” and each microstate can be stable for 80–120 ms before switching between different microstates. Microstate analysis differs from other methods in that signals from all electrodes are considered simultaneously, taking the global functional state into account. Microstate time series have a rich syntax that enables a variety of new quantifications of neurophysiologically relevant EEG signals. At the same time, the study of Lehmann et al. (2010) showed that the time series of EEG microstates will change with changes in behavior, disease, etc.

In previous studies, microstates were usually divided into four categories. Some researchers found that different microstates correspond to different brain regions in the human brain and reflect changes in different brain networks. Microstate A is primarily associated with the bilateral superior and middle temporal gyri, which are associated with the auditory system. Some studies showed that this area is closely related to the auditory network, reflecting the input and processing of auditory information (Britz et al., 2010). Microstate B is related to brain areas related to visual processing and may be connected to the visual network. Changes in microstate B are the first to be noticed when the human visual system is damaged or changed (Khanna et al., 2015). Microstate C is related to the posterior part of the anterior cingulate cortex, the bilateral inferior frontal gyrus, and the right anterior insula. It corresponds to the salience network and plays an important role in switching between the central executive network and the default network (Khanna et al., 2015). Microstate D is associated with locations such as the right dorsal and ventral areas of the frontoparietal cortex and with the central executive network, which is responsible for higher-level tasks such as cognition and decision-making (Khanna et al., 2015).

There is no report on the real-time monitoring of EEG during HBO therapy in patients with DOC. We believe that some EEG indicators of DOC patients will change during HBO therapy, and the characteristics of these changes may be helpful for the assessment of clinical consciousness level. Therefore, the purpose of this study was to observe the electrophysiological changes in patients with DOC before and during treatment by using the microstate change indicators of EEG. By analyzing the microstate indicators of patients, the feasibility of real-time EEG detection in HBO therapy and its role in brain function evaluation are herein discussed. EEG microstate changes during HBO therapy are likely to become important electrophysiological indicators for consciousness assessment.

## 2. Materials and methods

### 2.1. Patients

For this study, 41 DOC patients were recruited at the Beijing Tiantan Hospital, Capital Medical University, from March 2021 to January 2022. The age of the 41 DOC patients ranged from 18 to 76 years (47.7 ± 16.3), including 28 male patients (age 47.3 ± 16.9 years) and 13 female patients (age 48.8 ± 15.5 years). The postinjury period was 1–16 months (4.2 ± 3.8), and the CRS-R score before HBO therapy was 3–15 (7.1 ± 2.9) (Table 1). All enrolled patients met the following inclusion criteria: (1) definitively diagnosed with DOC; (2) age 18–80 years; (3) onset time more than 1 month; (4) consciousness in a stable period, unconscious improvement or decline at least 4 weeks before enrollment; and (5) family members who agreed to receive HBO therapy and signed an informed consent form.

**Table 1:** Details of DOC patients participating in real-time EEG monitoring during HBO therapy.

Patient	Gender	Age (years)	Etiology	Post-injure (months)	CRS-R						
					Total	A	V	M	OM	C	Ar
1	M	76	T	1.0	5	0	1	1	1	0	2
2	F	36	A	2.0	7	1	1	2	1	0	2
3	M	33	S	4.0	5	1	0	1	1	0	2
4	M	35	S	2.0	10	1	3	3	1	0	2
5	M	56	S	1.0	3	0	0	2	1	0	0
6	M	22	T	1.0	8	1	1	3	1	0	2
7	F	58	T	3.0	9	1	3	2	1	0	2
8	F	31	T	8.0	10	1	3	3	1	0	2
9	M	32	S	4.0	4	0	0	2	1	0	1
10	M	65	T	7.0	4	0	1	0	1	0	2
11	F	62	T	4.0	6	1	1	1	1	0	2
12	F	52	S	12.0	5	1	0	2	1	0	1
13	M	35	T	2.0	8	1	1	3	1	0	2
14	F	53	S	1.8	7	1	3	2	1	0	0
15	M	39	S	3.0	5	0	0	2	1	0	2
16	M	47	A	1.0	7	1	1	2	1	0	2
17	F	39	T	1.0	15	3	4	5	1	0	2
18	F	65	T	2.0	5	1	1	2	1	0	0
19	M	43	T	4.0	5	0	0	2	1	0	2
20	M	46	T	5.0	5	0	1	1	1	0	2
21	F	34	T	2.0	14	3	4	4	1	0	2
22	M	30	S	1.0	5	1	0	2	1	0	1
23	M	72	S	2.0	8	1	1	3	1	0	2
24	M	58	S	3.0	7	0	3	1	1	0	2
25	M	33	T	4.0	7	1	1	2	1	0	2
26	M	53	T	2.0	11	2	3	3	1	0	2
27	M	64	S	7.0	8	1	3	1	1	0	2
28	M	56	T	6.0	10	1	3	3	1	0	2
29	F	56	T	12.0	4	0	0	2	0	0	2
30	M	34	A	1.0	6	1	0	2	1	0	2
31	M	71	S	4.0	8	2	1	2	1	0	2
32	M	56	S	2.0	12	1	3	4	1	0	3
33	F	18	T	1.7	9	1	3	2	1	0	2
34	M	21	T	3.0	4	0	0	1	1	0	2
35	F	62	T	11.0	3	0	0	2	1	0	0
36	M	59	S	13.0	7	1	1	2	1	0	2
37	M	67	S	1.7	3	0	0	1	1	0	1
38	M	34	A	2.0	11	1	3	3	2	0	2
39	F	68	S	6.0	10	1	3	3	1	0	2
40	M	67	T	2.0	5	0	1	1	1	0	2
41	M	19	S	16.0	8	1	1	2	2	0	2

Sex (F, female; M, male); Etiology (A, anoxic; T, traumatic brain injury; S, stroke); CRS-R, Coma recovery scale-revised (A, auditory function; V, visual; M, motor; OM, oromotor; C, communication; Ar, arousal). Forty-one patients were included in the study, aged 18–76 years (47.7 ± 16.3), 1–16 months (4.2 ± 3.8) post-injury, and CRS-R score 3–15 (7.1 ± 2.9). Twenty eight male patients (age: 47.3 ± 16.9) and 13 female patients (age: 48.8 ± 15.5) were included in the study.

The exclusion criteria were as follows: (1) patients who could not tolerate HBO therapy, DOC caused by neurodegenerative diseases (Alzheimer’s disease, Lewy body dementia) and malignant brain tumor surgery; (2) coma caused by exacerbation of systemic diseases or expected survival time; (3) duration of illness <3 months; (4) epileptic seizures that were difficult to control; (5) treatment with experimental drugs or equipment; and (6) untreated tension pneumothorax or other conditions that HBO physician deems inappropriate for treatment.

The study was conducted under the Declaration of Helsinki of the World Medical Association and approved by the Ethics Committee of Beijing Tiantan Hospital (No. KYSQ 2021–396-01). Before inclusion, the researcher fully informed each patient’s legal guardians of the study protocol and obtained informed consent from them.

### 2.2. Clinical evaluation

During the period of HBO therapy, the level of consciousness of the patients was evaluated based on the CRS-R scale (Giacino et al., 2004). Each CRS-R score was performed independently by two trained clinicians. At least three assessments by CRS-R were performed 1 week before HBO therapy to clarify the patient’s level of consciousness.

### 2.3. HBO

The HBO therapy in this study was completed in the intensive care cabin group of the Hyperbaric Oxygen Department of Beijing Tiantan Hospital, Capital Medical University. The treatment pressure was 0.2 MPa (normal environment 0.1 MPa), and a single treatment lasted for 2 h: 30 min boost–60 min stabilized oxygen inhalation–30 min decompression. The oxygen chamber was equipped with a ventilator, sputum suction device, etc. ECG monitoring was maintained, and medical staff accompanied the whole process to ensure the safety of patients. During the treatment, the oxygen and carbon dioxide concentrations in the cabin were monitored to maintain them within the normal range.

### 2.4. EEG recording

EEG recordings were collected by Nicolet EEG with 19 channels. Data were collected synchronously during HBO therapy. The sampling rate was 500 Hz. The impedance of all electrodes was kept within 5 kΩ. During data collection, the bilateral mastoid electrodes A1 and A2 were used as reference electrodes.

### 2.5. EEG processing

We preprocessed the data using the eeglab plugins in the MATLAB toolbox. First, the data were filtered. A 50 Hz notch filter and 1–45 Hz bandpass filter were applied to the data according to the acquisition situation, some unnecessary electrodes (including bilateral mastoid electrodes A1 and A2) were removed, and the data were divided into 3 s epochs. Then, we removed the artifacts from the signal. First, most of the artifacts were removed by visual inspection, and then an independent component analysis (ICA) algorithm was used for correction to retain the characteristics of the original EEG signal but remove the artifacts associated with electrooculographic, electromyographic, and ECG events.

### 2.6. EEG microstate

To analyze the microstate of the preprocessed data, we first calculated its global field power (GFP). GFP can reflect the instantaneous electric field strength of the brain, so it is often used to measure the brain’s response to events or describe changes in brain activity (Khanna et al., 2015). The calculation formula of GFP is as follows:


$$GFP=\sqrt{\left(\overset{K}{\underset{i}{\sum }}{\left(V_{i}\left(t\right)-V_{mean}\left(t\right)\right)}^{2}\right)/K}$$

where K is the number of electrodes in the EEG data. $V_{i}\left(t\right)\;$is the potential of the i-th electrode at a certain time point. V_mean_(t) is the average value of the instantaneous potential across electrodes, and the formula is as follows:


$$V_{mean}\left(t\right)=\left(\overset{K}{\underset{i}{\sum }}V_{i}\left(t\right)\right)/K$$

In the microstate analysis, the topographic map of the local maximum value of GFP is considered to represent the discrete state of EEG. Therefore, through the cluster analysis of the local maximum value of GFP, all topographic maps are divided into several types. K-means clustering is the most basic method employed in the MATLAB toolbox. The method starts with partitioning the EEG samples into a fixed number of clusters, to which the EEG samples are relocated in iterations, until an optimal cluster assignment has been achieved. The clustering results were four microstates, labeled A, B, C, and D, and most of the differences could be explained using the topographic maps of the four microstates. After clustering the microstate topography map, we calculated the microstate parameters of all patients, including mean microstate duration (MMD), ratio of total time covered (RTT), global explained variance (GEV), transition probability, mean occurrence, and mean GFP.

### 2.7. Statistical analysis

Microstate analysis was performed on the EEG of all patients before and after HBO therapy. We analyze patients from different perspectives: (1) all patients (41 cases); (2) CRS-R ≥ 8 (17 cases) and CRS-R < 8 (24 cases); (3) traumatic brain injury and non-traumatic brain injury (including anoxic and stroke); and (4) other factors: age, sex, and postinjury (months). This study compares the differences in EEG microstates between different clinical groups before and after HBO.

We used SPSS 25 software for statistical analysis. We tested the data for normality using the Shapiro–Wilk test. The Wilcoxon signed-rank test was used for non-normally distributed data. The paired sample t-test was used to compare the microstate differences before and after HBO therapy. *p* < 0.05 was considered statistically significant.

## 3. Results

### 3.1. HBO treatment in DOC patients

All 41 patients completed a single session of HBO therapy and EEG monitoring, and no adverse events, such as middle ear and pulmonary barotrauma, occurred. The patients were treated smoothly in the HBO chamber, and there was no sudden instability of respiratory and blood pressure, suffocation with excessive phlegm, epilepsy, fever, or other events.

### 3.2. Analysis of EEG microstates in DOC patients

**Figure 1:**
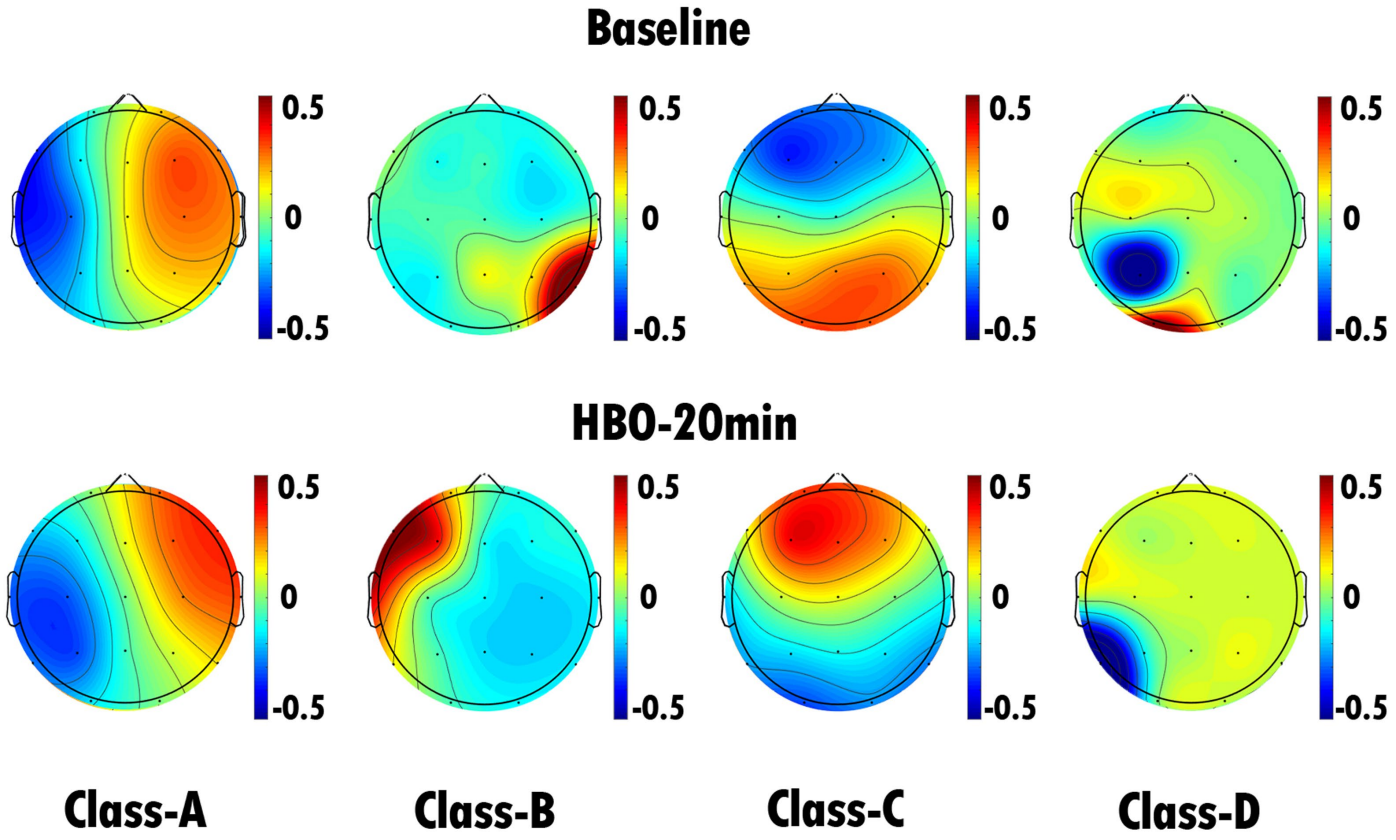
EEG microstate topography of all DOC patients (41 cases). The top row is the EEG microstate topography map of 41 DOC patients before HBO therapy, and the bottom row is the EEG microstate topography map of 41 DOC patients after 20 min of HBO therapy. Through statistical analysis, we found that the duration of microstate C in patients significantly increased after 20 min of HBO therapy compared with pretreatment (*p* < 0.05).

According to our previous research, when oxygen is inhaled for 20 min in a hyperbaric chamber, changes in patients’ EEG microstates can be observed, reflecting the impact of HBO on brain function (Yu et al., 2011, 2014). Referring to the research of Britz et al. (2010), we analyzed the EEG microstates of 41 patients with DOC by applying a clustering algorithm to fit four microstate topographic maps before HBO therapy and 20 min after HBO therapy; these microstates were designated A, B, C and D (Figure 1). The microstate class orientations were (A) right frontal left posterior; (B) left frontal right posterior; (C) anterior–posterior; and (D) frontocentral extreme (polarity is ignored in the microstate analysis).

**Figure 2:**
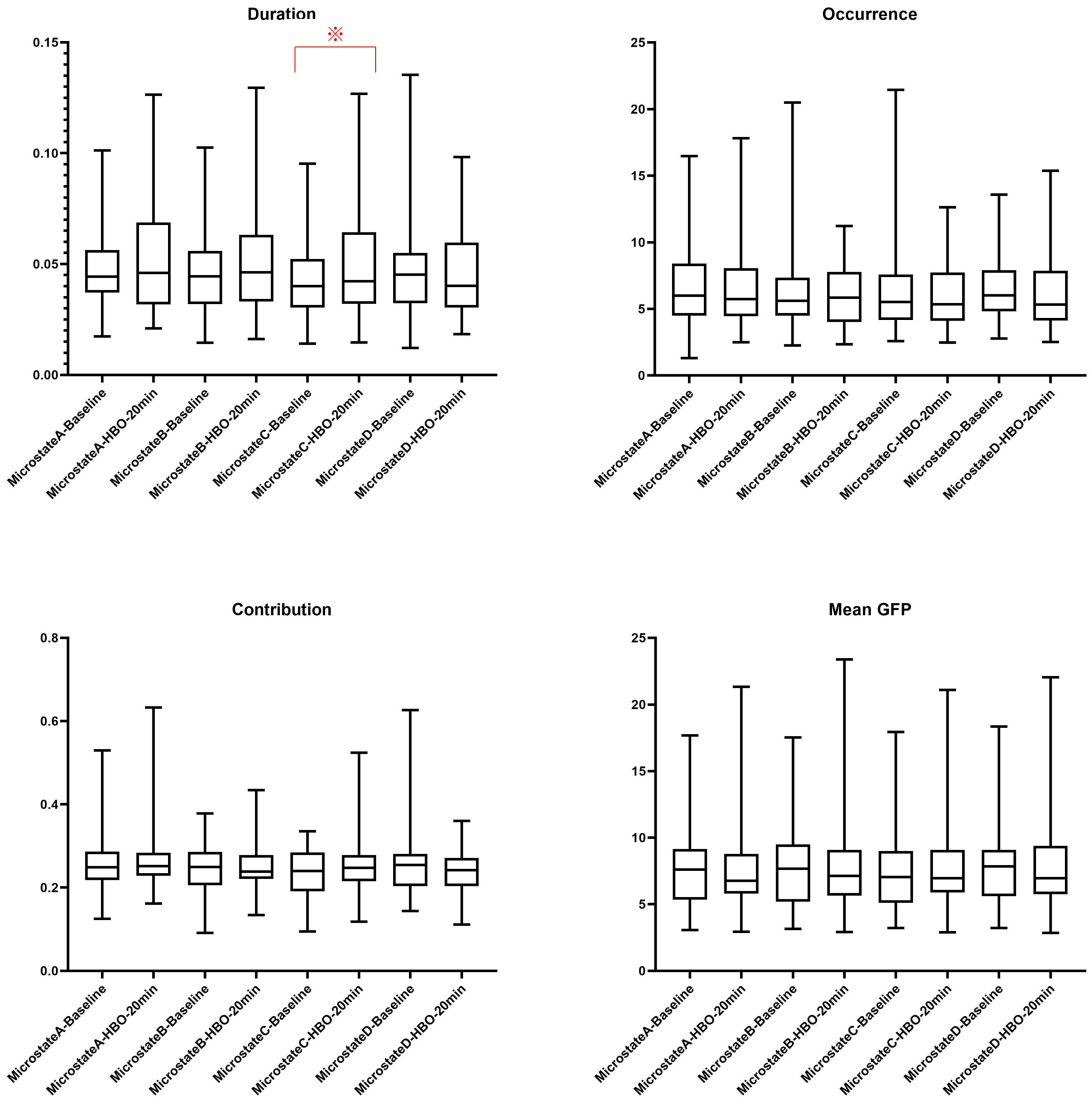
EEG microstate duration, occurrence, contribution and mean GFP analysis results of all DOC patients (41 cases). ^※^Indicates the *t*-test result was significant (*p* < 0.05).

Statistical analysis was performed on the changes in microstate parameters of 41 patients with DOC before and after HBO therapy. We found that the duration of microstate C in all patients at 20 min of HBO therapy was significantly increased compared with the corresponding pretreatment duration (*p* = 0.021) (Table 2 and Figure 2). The contribution, occurrence and mean GFP of all microstates did not change significantly. There was no significant change in the transition probabilities of the microstates in the 41 DOC patients (Table 3).

**Table 2:** EEG microstate duration, occurrence, contribution and mean GFP analysis results of all DOC patients (41 cases).

	Class A		Class B		Class C		Class D	
	Baseline (Mean ± SD)	HBO-20 min (Mean ± SD)	Baseline (Mean ± SD)	HBO-20 min (Mean ± SD)	Baseline (Mean ± SD)	HBO-20 min (Mean ± SD)	Baseline (Mean ± SD)	HBO-20 min (Mean ± SD)
Duration (s)	0.048 ± 0.021	0.053 ± 0.025	0.047 ± 0.021	0.050 ± 0.022	0.043 ± 0.018	**0.051 ± 0.025** ^**※**^	0.048 ± 0.024	0.047 ± 0.022
Occurrence (s^−1^)	6.614 ± 3.074	6.238 ± 2.793	6.417 ± 3.299	6.075 ± 2.401	6.783 ± 4.170	6.061 ± 2.497	6.558 ± 2.479	6.200 ± 2.880
Contribution (%)	0.258 ± 0.066	0.265 ± 0.077	0.248 ± 0.061	0.250 ± 0.065	0.236 ± 0.059	0.249 ± 0.065	0.259 ± 0.081	0.236 ± 0.051
Mean GFP (mV)	7.719 ± 3.206	8.018 ± 4.044	7.805 ± 3.343	7.994 ± 3.996	7.701 ± 3.427	8.002 ± 4.068	8.003 ± 3.314	7.967 ± 3.829

^※^Indicates the *t*-test result was significant (*p* < 0.05).

The duration of microstate C in 41 patients was significantly increased after 20 min of HBO therapy compared with the corresponding pretreatment duration (*p* < 0.05). Other microstate parameters did not change significantly.

**Table 3:** EEG microstate transition probability and GEV analysis results of all DOC patients (41 cases).

	Class A		Class B		Class C		Class D	
	Baseline (Mean ± SD)	HBO-20 min (Mean ± SD)	Baseline (Mean ± SD)	HBO-20 min (Mean ± SD)	Baseline (Mean ± SD)	HBO-20 min (Mean ± SD)	Baseline (Mean ± SD)	HBO-20 min (Mean ± SD)
Class A (%)	–	–	0.079 ± 0.025	0.083 ± 0.020	0.082 ± 0.024	0.086 ± 0.021	0.090 ± 0.031	0.084 ± 0.020
Class B (%)	0.081 ± 0.025	0.083 ± 0.020	–	–	0.081 ± 0.028	0.081 ± 0.018	0.081 ± 0.023	0.084 ± 0.023
Class C (%)	0.083 ± 0.026	0.087 ± 0.024	0.081 ± 0.026	0.081 ± 0.020	–	–	0.085 ± 0.027	0.081 ± 0.015
Class D (%)	0.087 ± 0.031	0.084 ± 0.021	0.084 ± 0.024	0.084 ± 0.023	0.086 ± 0.027	0.081 ± 0.015	–	–
GEV (%)	0.721							

The transition probability of all DOC patients did not change significantly.

### 3.3. Analysis of EEG microstates in DOC patients with different levels of consciousness

**Figure 3:**
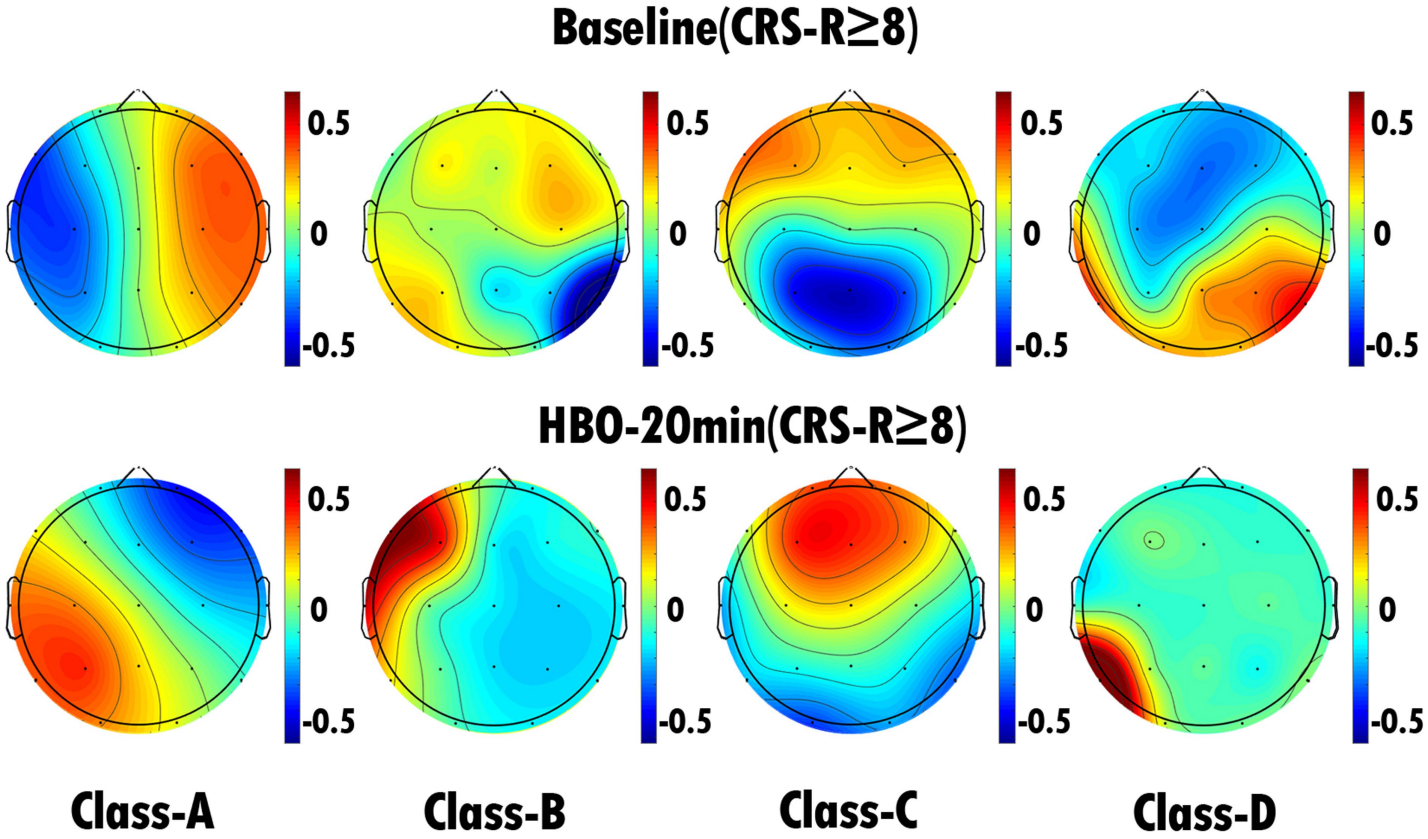
EEG microstate topography of DOC patients (17 cases) with CRS-R ≥ 8. The top row is the EEG microstate topography map of the patients before HBO therapy, and the bottom row is the EEG microstate topography map of the patients after 20 min of HBO therapy. We found that the duration of microstate C was significantly increased (*p* < 0.05) and the contribution of microstate D was significantly decreased (*p* < 0.05) after 20 min of HBO therapy compared with the pretreatment value.

**Figure 4:**
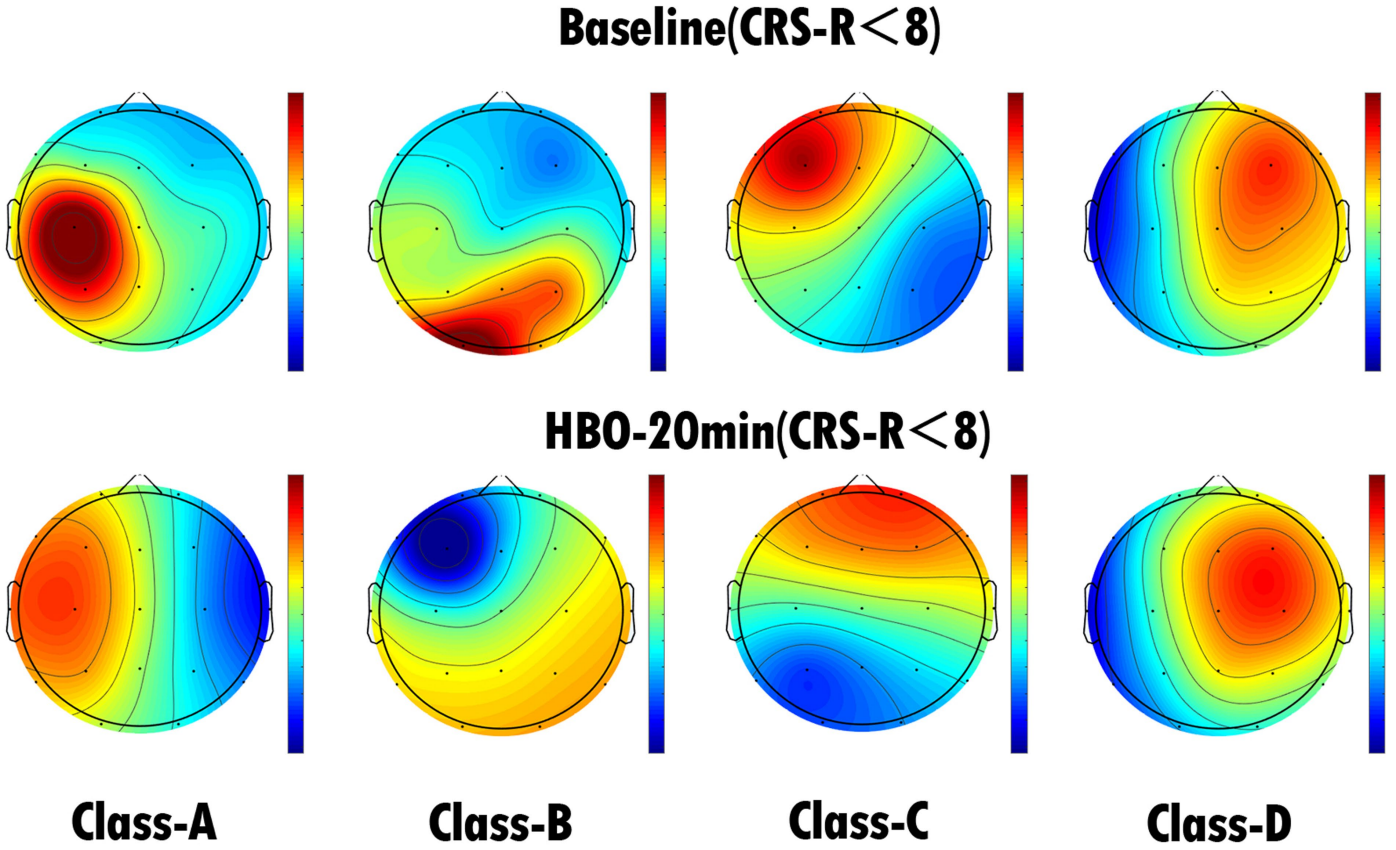
EEG microstate topography of DOC patients (24 cases) with CRS-R < 8. The top row is the EEG microstate topography map of the patients before HBO therapy, and the bottom row is the EEG microstate topography map of the patients after 20 min of HBO therapy.

**Figure 5:**
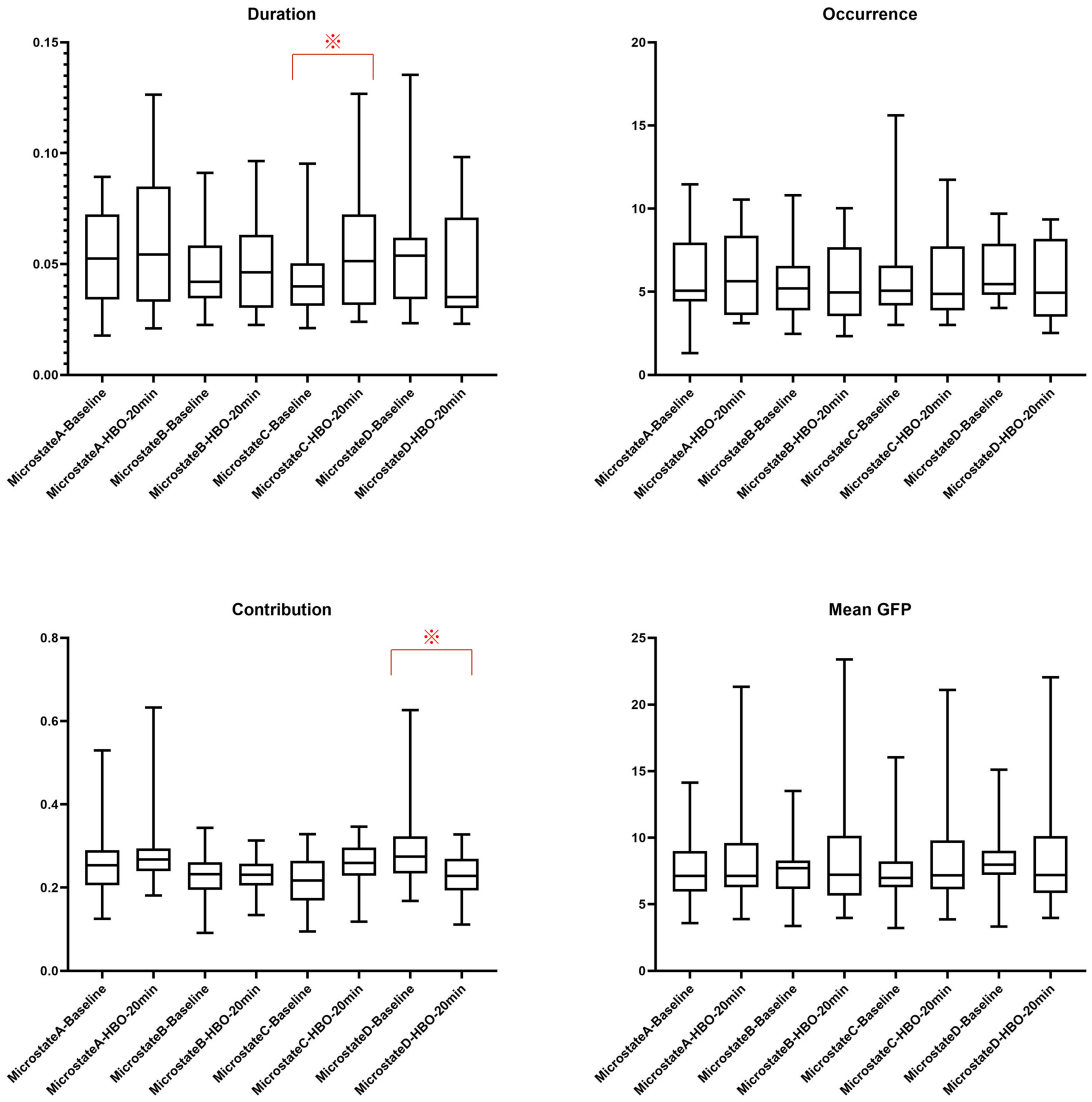
EEG microstate duration, occurrence, contribution and mean GFP analysis results of DOC patients (17 cases) with CRS-R ≥ 8. ^※^Indicates the *t*-test result was significant (*p* < 0.05).

**Figure 6:**
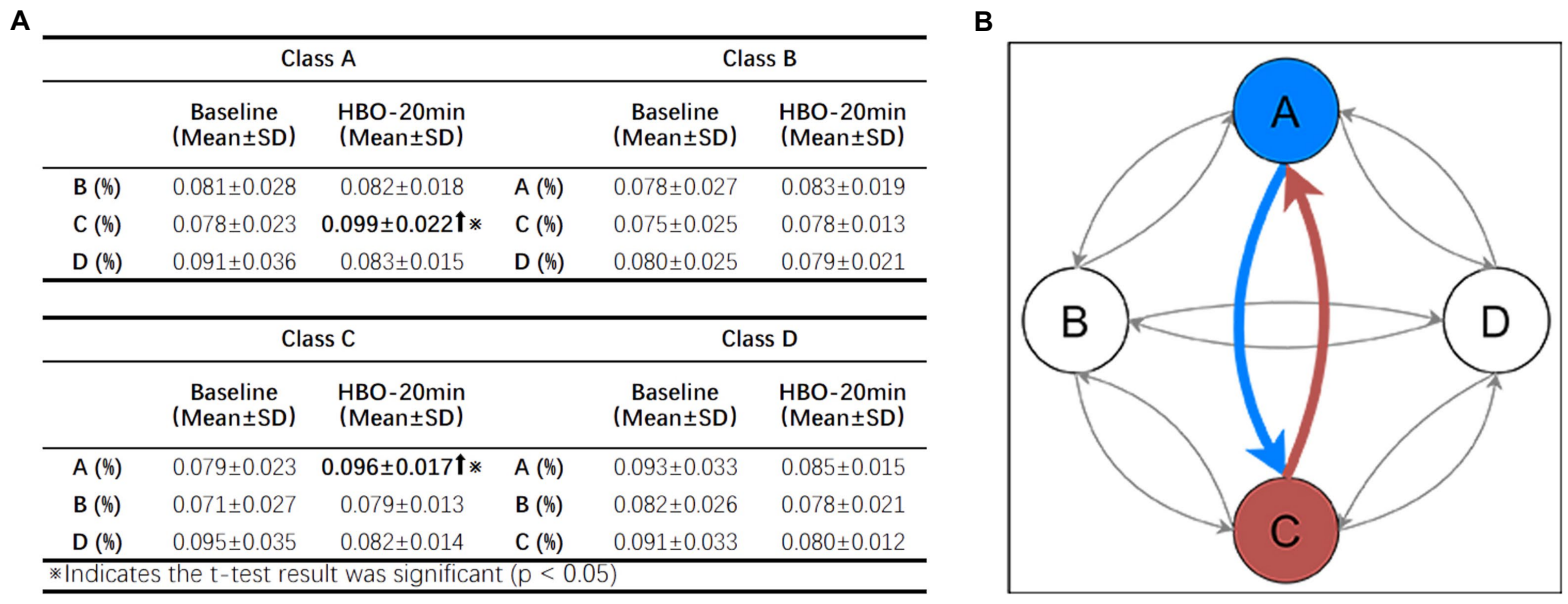
EEG microstate transition probability analysis results of DOC patients (17 cases) with CRS-R ≥ 8. **(A)** Analytical results of the EEG microstate transition probability in DOC patients (17 cases) with CRS-R ≥ 8. The transition probability from microstate A to microstate C and microstate C to microstate A was significantly increased after 20 min of HBO therapy compared with pretreatment (*p* < 0.05). **(B)** Schematic diagram of the mutual transition trends among the four microstates. The 4 solid circles represent 4 microstates, and the different arrows represent the transition trends between different microstates. The gray arrows represent that the transition trends between different microstates are not significant, and the bold colored arrows represent significant transition trends (*p* < 0.05).

We used the CRS-R scores to define the level of consciousness in DOC patients. We divided the patients into two groups by their CRS-R scores, namely, CRS-R ≥ 8 (17 cases) and CRS-R < 8 (24 cases), and performed a statistical analysis. The results showed that patients with CRS-R ≥ 8 had significant changes in microstates before and after HBO therapy (Figure 3), while those with CRS-R < 8 had no significant changes (Figure 4). The duration of microstate C in patients with CRS-R ≥ 8 at 20 min of HBO therapy was significantly increased compared with the pretreatment value (*p* = 0.047) (Table 4 and Figure 5). At the same time, the contribution of microstate D in these patients was lower at 20 min of HBO therapy than at pretreatment (*p* = 0.030). The results showed that the transition probability of microstates in patients with CRS-R ≥ 8 from microstate A to microstate C (*p* = 0.035) and microstate C to microstate A (*p* = 0.032) after 20 min of HBO therapy was significantly increased compared with the pretreatment probability. However, the transition probabilities among other microstates were not significantly different (Figure 6).

**Table 4:** EEG microstate duration, occurrence, contribution and mean GFP analysis results of DOC patients (17 cases) with CRS-R ≥ 8.

	Class A		Class B		Class C		Class D	
	Baseline (Mean ± SD)	HBO-20 min (Mean ± SD)	Baseline (Mean ± SD)	HBO-20 min (Mean ± SD)	Baseline (Mean ± SD)	HBO-20 min (Mean ± SD)	Baseline (Mean ± SD)	HBO-20 min (Mean ± SD)
Duration (s)	0.052 ± 0.022	0.060 ± 0.030	0.047 ± 0.020	0.049 ± 0.020	0.043 ± 0.018	**0.056 ± 0.029** ^**※**^	0.055 ± 0.026	0.050 ± 0.025
Occurrence (s^−1^)	6.227 ± 2.840	6.070 ± 2.360	5.623 ± 2.358	5.653 ± 2.389	6.086 ± 3.460	5.937 ± 2.482	6.161 ± 1.929	5.741 ± 2.421
Contribution (%)	0.266 ± 0.091	0.289 ± 0.102	0.228 ± 0.058	0.226 ± 0.048	0.217 ± 0.062	0.259 ± 0.053	0.289 ± 0.101	**0.227 ± 0.053** ^**※**^
Mean GFP (mV)	7.686 ± 2.587	9.304 ± 5.231	7.674 ± 2.539	9.121 ± 5.263	7.569 ± 2.963	9.324 ± 5.267	8.243 ± 2.782	9.162 ± 4.954

^※^Indicates the *t*-test result was significant (*p* < 0.05).

The bold values indicate the *t*-test result was significant (*p* < 0.05).

The duration of microstate C of 17 patients had significant increased after 20 min of hyperbaric oxygen therapy compared with pre-treatment (*p* < 0.05). And the contribution of microstate D had significant reduced (*p* < 0.05). Other microstate parameters did not change significantly.

### 3.4. Analysis of EEG microstates in patients with DOC with different etiologies

From the perspective of etiology, we divided the patients into two groups: traumatic brain injury and nontraumatic brain injury (including anoxic and stroke), and the microstate parameters of each group of patients were statistically analyzed. The results showed that the duration of microstate C in nontraumatic brain injury patients after 20 min of HBO therapy was increased compared with the pretreatment duration. The mean GFP of patients with traumatic brain injury showed some decrease after 20 min of HBO therapy compared with pretreatment. There were no significant differences in the remaining microstate parameters between the two groups.

Statistical analysis showed that there was no significant difference in the microstate parameters of patients according to age, sex, or postinjury time either before or after they received HBO therapy (*t*-test, *p* > 0.05).

## 4. Discussion

With the continuous development of HBO medicine, comprehensive rehabilitation treatment based on HBO can clinically benefit patients with DOC. As an important electrophysiological technique, EEG detection is widely used in the evaluation of brain function in DOC patients. It is safe and feasible to carry out real-time EEG monitoring research under HBO therapy, which can not only provide an objective basis for precise HBO therapy but also enrich the clinical evaluation indicators of DOC. In this study, through the microstate analysis of EEG signals before and during HBO therapy, we found that some microstate indicators of DOC patients were significantly different.

Since the recovery of consciousness is not only a dynamic process but also one that involves interaction among various regions of the brain (Lei et al., 2022), the improvement of the brain functional network precedes the change in behavioral signs of awareness in DOC patients (Bareham et al., 2020). It is speculated that HBO therapy restored the function of some brain cells and enhanced the connections among different brain regions. If HBO therapy is used as a stimulating condition, the EEG changes during HBO can be monitored under this condition, which may help clinicians assess the patient’s level of consciousness and formulate a more scientific clinical treatment plan.

In this study, we included 41 patients with different degrees of DOC, collected their baseline information, and completed real-time EEG monitoring during HBO therapy. Compared with previous DOC research (Stefan et al., 2018), our study included more patients with more extensive degrees of DOC, thus our results were more representative.

### 4.1. Research status of the microstate in DOC patients

Recently, many studies have shown that the microstate indicators of patients with neurological and psychiatric diseases will change accordingly and may become potential biomarkers of certain types of diseases (de Bock et al., 2020; Wang et al., 2021; Sun et al., 2022). Microstate analysis can also be used as one of the indicators of drug treatment effects in patients with certain diseases (Serrano et al., 2018). In recent years, EEG microstate analysis of DOC patients has also developed rapidly. Representative studies have shown that microstate indicators can provide new anchor points for DOC patient evaluation and DOC patient prognosis assessment. For example, Stefan et al. (2018) analyzed 63 DOC patients’ EEG, predicted the prognosis of 39 patients, and found that microstate A had significant differences. Guo et al. (2022) showed that DOC patients had significant changes in microstate C and microstate D after high-definition transcranial direct current stimulation (HD-tDCS) treatment. Zhang et al. (2022) performed microstate analysis on resting-state EEG data from DOC patients and identified seven microstates with distinct spatial distributions of cortical activation. There were significant differences in the microstate between the MCS group and the *VS* group. In existing research related to DOC, EEG data was collected from patients in the resting state for microstate analysis, but there is no related research on real-time EEG monitoring under special conditions. Our research on EEG real-time monitoring under resting-state and HBO conditions is of great significance for future studies on HBO treatment effects and disease indicators of DOC. Therefore, this study can not only greatly enrich real-time EEG monitoring in special environments but also provide new ideas for EEG research in DOC patients in an HBO environment.

### 4.2. Microstates of DOC patients during HBO therapy

Based on the EEG signals of all 41 DOC patients included in the study, we used a clustering algorithm to fit topographic maps to the microstates before and after 20 min of HBO therapy and divided them into four categories: A, B, C, and D (Figure 1). From the fitted topographic maps, we can see that microstate B and microstate D of DOC patients are quite different, while microstate A and microstate C conform to the classic microstate type compared with the four classic resting microstate topographic maps of normal people found by previous researchers (Britz et al., 2010). Through the statistical analysis of the microstate parameter results of all 41 DOC patients included in the study, we found that the duration of microstate C in patients after HBO therapy was generally significantly increased compared with the duration before treatment.

Microstate B is considered to be related to bilateral visual cortex regions. The topographic map of microstate B in DOC patients is quite different compared with that in normal individuals, which may be related to the changes in the vision-related brain network in DOC patients (Britz et al., 2010). However, due to the different basic conditions of patients at admission, more research is needed to verify this hypothesis. Before and after a single HBO treatment, the parameters related to microstate B of the patients did not change significantly, which suggests that 20 min of HBO therapy is not enough to cause EEG changes in the relevant brain regions of the patients.

Compared with the microstate parameters of patients before and after 20 min of HBO therapy, the parameters related to microstate C increased significantly. Although the physiological basis of microstate C is still highly controversial, in the study of Britz et al. (2010), microstate C was considered to be part of the salience network, which mainly detected and responded to internal or external stimuli received by other brain networks. The salience network, which plays a key role in switching between central executive functions and default modes, can combine interoceptive information with emotional salience to form subjective representations of one’s own body (Sun et al., 2022). As the “mediator” of the brain, the salience network plays an integral role in the processing of sensorimotor information, general cognition, and the coordination between emotion, pain, and body movement. The significant increase in the microstate C parameters of patients may indicate that brain areas related to high-level functions such as attention and cognition are activated during HBO therapy.

The microstate D of patients is also significantly different from the microstate map of normal people. In the current study, microstate D is considered to correspond to the central executive network of the brain, and the central executive network is related to functions such as task selection and decision-making and is responsible for high-level cognitive tasks. Through the analysis of the microstate, we found that the cognitive and decision-making-related cortical areas of DOC patients are in a state of inhibition, which is also consistent with the clinical diagnosis of DOC.

### 4.3. Microstates of DOC patients with CRS-R scores under HBO conditions

We fitted the microstate topographic maps of the 17 patients with CRS-R ≥ 8 before and after treatment (Figure 3). Similar to the results in Figure 1, the microstate B and microstate D of these 17 DOC patients were significantly different from those found by Britz et al. (2010). By analyzing the microstate parameters of 17 patients with CRS-R ≥ 8, we found that the duration of microstate C in patients with CRS-R ≥ 8 was increased significantly compared with the pretreatment duration; moreover, the contribution of microstate D decreased significantly, and the transition between microstate A and microstate C increased significantly compared with the pretreatment values.

In the current study, microstate A was thought to be associated with the activation of the bilateral superior and middle temporal gyri regions, which are associated with functions such as hearing. The transition probability between microstate A and microstate C in patients increased significantly, which may indicate that the input of auditory information in patients with CRS-R ≥ 8 became more active during HBO therapy than when they were not treated. This shows that HBO therapy may increase the information exchange between the auditory network and salience network in patients. Increased processing of auditory information may indicate some recovery from brain damage in patients. This type of result did not appear in patients with CRS-R < 8, which indicates that patients with a relatively better level of consciousness have better activation of the auditory network in response to HBO therapy. This is similar to recent findings by Guo et al. (2022), who reported that the probability of occurrence per second (OPS) of microstate D was positively correlated with CRS-R scores in *VS* and MCS patients before HD-tDCS treatment. Patients progress from *VS* to MCS when the OPS in microstate D increases. This may indicate that patients with higher CRS-R scores have better recovery of consciousness. Whether increasing the frequency of HBO therapy will affect similar changes in patients with CRS-R < 8 remains to be further studied.

## 5. Limitations

This study only analyzed the EEG detection data during a single session of HBO therapy. Some indicators were found to be significantly different in the EEG microstate, but the results were not enough to explain the effectiveness of HBO therapy, especially the superposition of multiple HBO therapy effects. Further research is still needed to confirm this hypothesis. In addition, this was an exploratory, single-center study with relatively large limitations, and the results should be interpreted with caution. The number of included samples is not sufficient, and the etiologies of the DOC cases are extremely heterogeneous. The results of EEG microstate changes under HBO are not sufficient to explain the brain network response of DOC patients’ consciousness from the level of pathophysiological mechanism.

## 6. Conclusion

Real-time EEG detection in DOC patients during HBO therapy is safe and feasible. The results of this study show that HBO therapy has a specific activating effect on attention and cognitive control in patients and causes increased activity in the primary sensory cortex (temporal lobe and occipital lobe). This study demonstrates that real-time EEG detection and analysis during HBO is a clinically feasible method for assessing brain function in patients with DOC. During HBO therapy, some EEG microstate indicators show significant changes related to the state of consciousness in patients with prolonged DOC. This will be complementary to important electrophysiological indicators for assessing consciousness and may also provide an objective foundation for the precise treatment of patients with DOC, but more research is needed.

## Data availability statement

The raw data supporting the conclusions of this article are available on request to the corresponding authors.

## Ethics statement

The studies involving human participants were reviewed and approved by IRB of Beijing Tiantan Hospital, Capital Medical University. The legal guardians of the patients with chronic disorders of consciousness provided their written informed consent to participate in this study.

## Author contributions

JW, LX, QY, and XZ conceived and designed the research. LC, XG, XC, LX, YLL, and CW performed the experiments. YW, YL, and JW were responsible for the data analysis. QG, YZ, JW, LX, QY, JH, and XZ drafted the manuscript. All authors had a lot of contributions at all stages of preparing the manuscript.

## Funding

This work was supported in part by the National key research and development program of China (grant no. 2021ZDZD0204200), the Ministry of Science and Technology of China (grant nos. 2022ZD0211901, 2019YFA0707103, 2020AAA0105601), the National Nature Science Foundation of China (grant nos. 31730039, U21A20388), and the Chinese Academy of Sciences grants (grant no. ZDBS-LY-SM028).

## Conflict of interest

The authors declare that the research was conducted in the absence of any commercial or financial relationships that could be construed as a potential conflict of interest.

## Publisher’s note

All claims expressed in this article are solely those of the authors and do not necessarily represent those of their affiliated organizations, or those of the publisher, the editors and the reviewers. Any product that may be evaluated in this article, or claim that may be made by its manufacturer, is not guaranteed or endorsed by the publisher.
